# The risk of refracture and malunion in children treated for diaphyseal forearm fractures: a retrospective cohort study

**DOI:** 10.2340/17453674.2025.42851

**Published:** 2025-02-24

**Authors:** Hans-Christen HUSUM, Ole RAHBEK, Per Hviid GUNDTOFT, Hans Christian BANG, Søren KOLD, Jan Duedal RÖLFING, Ahmed ABOOD

**Affiliations:** 1 Interdisciplinary Orthopedics, Aalborg University Hospital, Aalborg; 2 Department of Orthopedics, Aarhus University Hospital, Aarhus, Denmark

## Abstract

**Background and purpose:**

The optimal treatment modality for pediatric diaphyseal forearm fractures is debated. While nonoperative treatment and closed reduction reduces the need for surgery and surgical complications, flexible intramedullary nailing (FIN) may reduce refracture and malunion rates. We aimed to compare the relative risk (RR) of refracture and malunion between nonoperative, closed reduction (CR), and surgical treatment in children treated for diaphyseal forearm fractures.

**Methods:**

We performed a retrospective cohort study of children treated for a primary diaphyseal forearm fracture over a 9-year period at 2 university hospitals. Risk of refracture and malunion in the year following the fracture across treatment modalities was assessed by a modified Poisson regression while adjusting for the age of the patient at the time of fracture.

**Results:**

We included 837 patients for analysis, of whom 4% were treated nonoperatively, 6% were treated with closed reduction, and 90% with FIN. Compared with FIN, the RR of refracture was higher for the nonoperative group (9.8, 95% confidence interval [CI] 5.9–16.3) and CR group (2.5, CI 1.2–5.3). Compared with the FIN group, the nonoperative and CR groups had higher risk of malunion with RR of 15.3 (CI 11.0–21.4) and 8.5 (CI 5.8–12.5) respectively. Of those treated non-surgically, 84% remained without any surgery. In FIN patients, surgical revision due to infection was seen in 1.4% of patients.

**Conclusion:**

The risk of refracture and malunion in children treated for diaphyseal forearm fractures was significantly higher for closed reduction and nonoperative treatment compared with FIN treatment.

Nonoperative treatment, closed reduction (CR), and flexible intramedullary nailing (FIN) are the most common employed treatment approaches in pediatric diaphyseal forearm fractures. While nonoperative treatment consists of applying a cast to fractures in near anatomical alignment, CR involves manipulating the fractured bones back into alignment. CR is particularly used in younger children because their fractures need not to be anatomically reduced as their bones retain significant remodeling potential [1]. However, as children age beyond 8–10 years, their remodeling capacity reduces, increasing the likelihood of malunion that may lead to permanent impaired function [2,3]. Remodeling potential is reflected in treatment guidelines. The most referenced guideline [4] is based on expert opinion, given the sparse evidence in the field at the time. A recent systematic review [5] found no significant advancements in knowledge since these recommendations from 1998 and no high-quality prospective studies are available.

CR offers the advantage that it can be performed in emergency department or outpatient settings, potentially reducing healthcare costs associated with operating room utilization and surgical implants [5]. However, it requires personnel experienced in pediatric sedation and pain management, fracture reduction, and application of a stable cast [1]. This level of expertise in the emergency department is not readily available in all healthcare settings. A disadvantage of CR is reported to be a 30% risk of surgical intervention due to re-displacement of the fracture [6].

FIN treatment has demonstrated a significant reduction in malunion rates compared with CR and is used increasingly [7,8]. FIN is complicated, with nerve deficits in up till 10% of cases. Most nerve deficits are, however, transient with 3% being permanent [9,10]. Long-term follow up of FIN treatment has been reported with favorable patient-reported outcomes [11]. However, if FIN removal is chosen, this procedure incurs associated costs [5] and potential complications of a second surgery [12,13].

The treatment-dependent risk of refracture is a crucial consideration in selecting the optimal treatment strategy for diaphyseal forearm fractures in children. The incidence of refractures ranges between 2% and 8% [5,6,11,14-16]. However, reliable data on refractures is sparse.

This retrospective cohort study aims to compare the relative risks of refracture and malunion in children treated for diaphyseal forearm fractures. Moreover, surgical procedures were registered for each treatment group.

## Method

### Study design

This is a retrospective cohort study of children with diaphyseal forearm fractures treated nonoperatively, with CR, or with FIN at Aalborg University Hospital (AAUH) and Aarhus University Hospital (AUH) from January 2012 to January 2021. Reporting follows the STROBE guidelines for reporting on observational studies [17].

### Participants

We included children (females under 14 years of age and males under 16 years of age), treated for a primary diaphyseal forearm fracture (i.e., not a refracture). Patients were identified using ICD-10 diagnosis codes identifying diaphyseal forearm fractures (DS522, DS523, DS524, DS526, DS527, DS528, DS529) and Nomesco Classification of Surgical Procedures (NCSP) codes (KNCJ01, KNCJ04, KNCJ06, KNCJ08, KNCJ 41, KNCJ 44, KNCJ46, KNCJ50, KNCJ51, KNCJ52, KNCJ53, KNCJ54, KNCJ55, KNCJ56, KNCJ57, KNCJ58, KNCJ91, KNCJ91, KNCJ96).

We excluded Monteggia and Galeazzi fractures and patients with refractures that did not have a primary fracture within the study period, patients without follow-up (e.g., tourists) or less than 1-year of follow-up, and fractures treated with Kirschner wire or plates.

### Variables

The collected variables for each included patient were: basic demographic information, the date of fracture, type of procedures, procedure dates, cast treatment type (forearm/above-elbow), duration of immobilization in a cast, and refractures. Time immobilized by cast was counted only for the primary treatment, thus immobilization time resulting from treatment conversion or additional surgical intervention due to complications was not counted.

All included fractures were classified and re-measured at the time of inclusion by co-author HB and a medical student. A diaphyseal fracture was classified by a fracture located in the diaphysis of either the radius or the ulna, where the diaphysis was defined according to the AO foundation. There were no restrictions on whether patients in the FIN group had their intramedullary nails removed or not at the final follow-up. Both raters had received instruction in the measurement of angulation and displacement in the sagittal and frontal plane of each included fracture using the Picture Archiving and Communications System (PACS). Initial measurements were validated by the senior author AAA to ensure the quality of measurements. Malunion of fractures was defined as an angulation ≥ 10° in the sagittal or frontal plane [5] and/or a displacement of ≥ 50% of the bone width at the final follow-up radiograph examination [18]. We defined refracture as a fracture occurring spontaneously or following trauma at the same location within 1 year after the initial fracture.

### Data sources

Data was collected through review of electronic patient charts and radiographic images in PACS. Data was manually entered and stored in a shared encrypted Excel file (Microsoft Corp, Redmond, WA, USA) within the participating institutions’ secure digital infrastructure.

### Statistics

Descriptive continuous variables are presented as means and standard deviations (SD) if normally distributed, otherwise medians and interquartile range (IQR) are given. Comparisons were made using chi-square; ANOVA and Kruskal–Wallis tests were applied for both descriptive and outcome variables for comparison of categorical outcomes, means, and medians respectively.

As we included only patients with 1-year of follow-up, or who were followed until an event, in the analysis of risk of refractures, relative risk (RR) of refracture and malunion across treatment groups was calculated using a modified Poisson regression and survival estimates were visually presented as Kaplan–Meier curves. The analysis was performed with each patient grouped by their primary treatment allocation, regardless of later conversion in treatment. For instance, a patient initially treated with CR who was converted to FIN treatment due to secondary dislocation would be registered as a refracture in the CR group regardless of whether the refracture occurred after treatment conversion [19].

To investigate any confounding effect of age and sex on risk of refracture, the relative risk of refracture stratified by treatment type was calculated as a crude, age, and sex-adjusted estimates.

Normal distribution of data was inspected using Q–Q plots and a 5% significance level was applied. All calculations were performed using Stata version 17.0 (StataCorp LLC, College Station TXs, USA).

### Ethics, registration, data sharing plan, funding, use of AI, and disclosures

The study was approved by the relevant ethics committee at each institution (registration numbers 2021-005755 and 1-45-70-11-21). No external funding was received in the planning or conducting of this study. Due to the sensitive nature of the study data, data is not freely accessible, but a sharing agreement can be obtained by written contact with the authors. AI was not used in the preparation of this manuscript or in the analysis of data.

Complete disclosure of interest forms according to ICMJE are available on the article page, doi: 10.2340/17453674.2025.42851

## Results

### Participants

**Figure 1:**
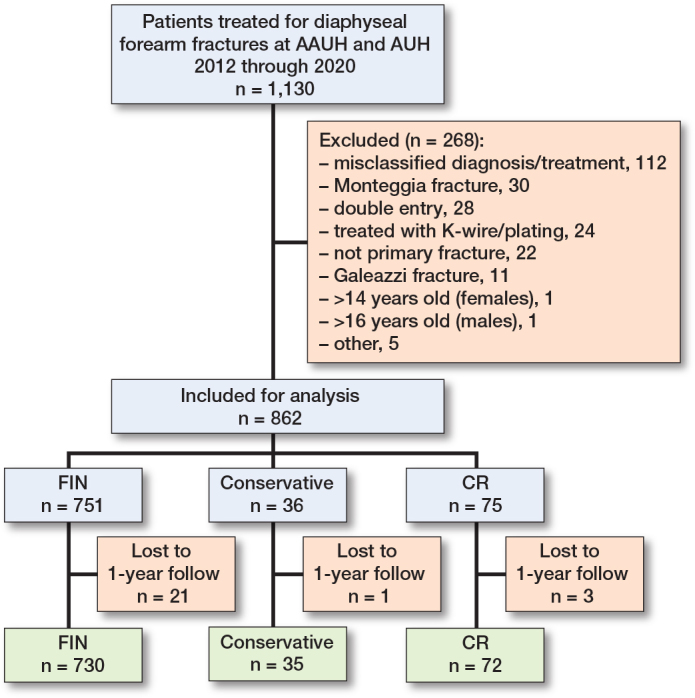
Flow diagram of the inclusion process. CR = closed reduction, FIN = flexible intramedullary nail. AAUH = Aalborg University Hospital, AUH = Aarhus University Hospital.

We identified 1,130 patients treated for diaphyseal forearm fracture at our institutions within the study period. 268 of these were excluded, primarily due to misclassification of diagnosis and/or treatment (n = 112), definitive treatment performed at another institution (n = 34), and Monteggia/Galeazzi fractures (n = 30) (Figure 1). 3 patients were missing the correct radiographic projections for malunion analysis, and 41 records were missing dates of cast removal, all in the FIN group. 25 patients were lost to follow-up after definitive treatment. 21 of these (84%) were treated with FIN, 3 (12%) with CR, and 1 (4%) was treated nonoperatively. Data from 837 patients was available for analysis. Of these, 35 (4%) were treated nonoperatively, 72(9%) were treated with CR, and 730 (87%) were treated with FIN.

Compared with the mean age of patients in the FIN group (8.7 years, CI 8.5–8.9), the mean age of patients in the nonoperative group (7.4 years, CI 6.1–8.7) and CR group (5.9 years, CI 5.2–6.7) was lower at –1.3 years (CI –2.4 to –0.2) and –2.8 years (CI –3.6 to –2.0) respectively (Table 1).

**Table 1:** Baseline characteristics for the 3 treatment groups. Values are count (%) unless otherwise specified

Factor	Nonoperative	CR	FIN	Total	P value
Individuals	35	72	730	837	
Male sex	17	45	411 (56)	473 (57)	0.4
Age, mean (SD)	7.4 (4)	5.9 (3)	8.7 (3)	8.4 (3)	< 0.001
Fracture location					0.003
Radius + ulna	35	71	610 (84)	716 (86)	
Radius	0	0	92 (13)	92 (11)	
Ulna	0	1	28 (4)	29 (4)	
Cast type					< 0.001
No cast	0	0	82 (11)	82 (10)	
Forearm	1	0	133 (18)	134 (16)	
Above elbow	34	72	515 (71)	621 (74)	
Casting (days), mean (SD)	36 (22)	32 (7)	23 (11)	24 (11)	< 0.001
Converted to FIN treatment	11	6	N/A	17 (16)	< 0.001

CR = closed reduction, FIN = flexible intramedullary nail,

SD = standard deviation.

P values are calculated using ANOVA tests, Kruskal–Wallis tests or chi-square tests for means, medians, and proportions respectively.

While there were 120 1-bone fractures (16%) and 133 (18%) forearm casts in the FIN group, all but 1 case were 2-bone fracture and above-elbow casts in the CR and nonoperative groups. Compared with the mean immobilization time of the FIN group of 22 days (CI 22–23), the mean immobilization time for the nonoperative group (36 days, CI 27–45) and CR group (32 days, CI 30–34) was significantly higher at 13 days (CI 9–18) and 10 days (CI 7–12) respectively.

### Outcomes

#### Refractures

During 1 year of follow-up, 55 patients (7%) experienced a refracture. Compared with 4% (CI 3–6) of FIN patients, the proportion of patients experiencing a refracture in the nonoperative (43%, CI 27–59) and CR groups (11%, CI 4–18) was higher by 39% (CI 22–55) and 7% (CI 0–14), respectively (Table 2). Of the 32 refractures in the FIN group, 11 patients had their FIN removed before refracture occurred.

**Table 2:** Refractures, malunions, and relative risks for patients treated either nonoperatively or with closed reduction (CR) compared with treatment with flexible intramedullary nail (FIN)

	FIN	Nonoperative	CR	P value
Refractures, [n] % (CI)	[32] 4.4 (2.9–5.9)	[15] 43 (27–59)	[8] 11 (3.9–18)	< 0.001
Malunion, [n] % (CI)	[41] 5.7 (4.0–7.4)	[21] 88 (74–100)	[32] 49 (36–74)	< 0.001
Relative risk of refracture				
crude	–	9.8 (5.9–16.3)	2.5 (1.2–5.3)	< 0.001
adjusted	–	10.0 (6.0–16.8)	2.5 (1.2–5.5)	< 0.001
Relative risk of malunion				
crude	–	14.3 (10.1–20.2)	7.4 (4.9–11.4)	< 0.001
adjusted	–	12.8 (8.4–19.5)	6.9 (4.4–10.9)	< 0.001

Adjusted relative risks were adjusted for age at fracture and sex.

CI = 95% confidence interval; CR = closed reduction; FIN = flexible intramedullary nail.

**Figure 2:**
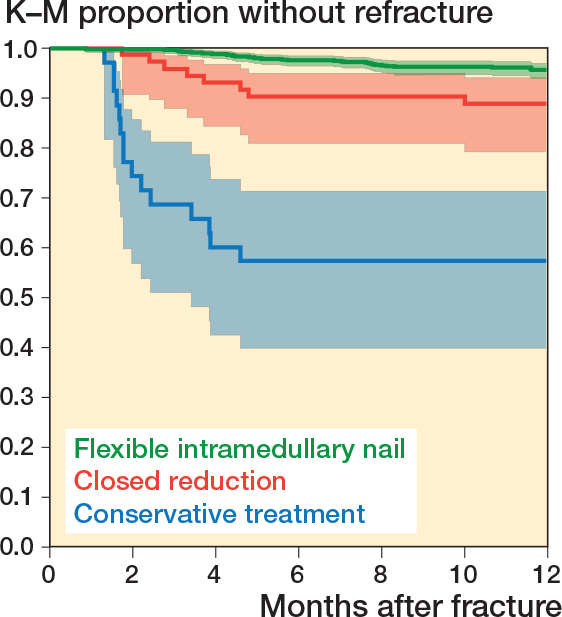
Survival curves and accompanying 95% CI by treatment of refractures grouped by primary treatment allocation within a 1-year follow-up period. Blue = nonoperative treatment, red = closed reduction, green = flexible intramedullary nail.

Compared with the FIN group, there was a significantly higher risk of refracture in the nonoperative group with an RR of 9.8 (CI 5.9–16.3) and in the CR group with an RR of 2.5 (CI 1.2–5.3). Adjusting for age at time of primary fracture and sex did not influence the calculated relative risk of refracture across treatment groups. Additionally, compared with the FIN group, time to refracture was shorter in the nonoperative and CR groups (Figure 2, Table 1).

#### Malunions and complications

At the time of final follow-up radiograph, malunion was present in 94 (12%) of the total number of patients. Compared with the 6% (CI 4–7) of FIN patients, the proportion of patients experiencing a malunion in the nonoperative (88%, CI 74–100) and CR groups (49%, CI 36–74) was higher by 82% (CI 68–95) and 43% (CI 31–55), respectively (Table 2).

Compared with the FIN group, the nonoperative and CR groups had a higher risk of malunion with an RR of 15.3 (CI 11.0–21.4) and 8.5 (CI 5.8–12.5), respectively. Adjusting for age at the time of fracture, sex, and interval between primary and final radiograph did not affect this result. There were no detectable differences in malunion across age groups for all treatment modalities (Table 3).

**Table 3:** Malunion and days from primary to final radiograph of patients who were not converted to other treatment modalities stratified by age groups

	0–5 years n = 124	5–10 years n = 358	> 10 years n = 355	Total N = 837	P value
FIN					
Individuals, n (%)	86 (12)	312 (43)	332 (46)	730 (100)	
Malunion at final radiographic examination, n (%)	8 (9)	18 (6)	17 (5)	43 (6)	0.3
Days from primary to final radiograp, median (IQR)	49 (29–93)	60 (35–102)	85 (44–122)	75 (40–112)	< 0.001
Nonoperative					
Individuals, n (%)	10 (29)	15 (43)	10 (29)	35 (100)	
Malunion at final radiograph examination, n (%)	10 (100)	14 (93)	8 (80)	32 (91)	0.3
Days from primary to final radiograph, median (IQR)	12 (8–22)	16 (8–32)	29 (9–36)	16 (8–30)	0.3
CR					
Individuals, n (%)	28 (39)	31 (43)	13 (18)	72 (100)	
Malunion at final radiograph examination, n (%)	13 (46)	20 (65)	5 (39)	38 (53)	0.2
Days from primary to final radiograph, median (IQR)	30 (24–32)	31 (24–36)	41 (32–102)	30 (25–39)	0.01

IQR = interquartile range. CR = closed reduction, FIN = flexible intramedullary nail.

During the study period, 11 (31%) patients in the nonoperative group and 6 (8%) in the CR group were converted to FIN treatment due to loss of reduction. Of those treated primarily with FIN, 10 (1%) patients had infections, which required further surgical intervention.

## Discussion

We compared the relative risk (RR) of refracture and malunion between nonoperative, CR, and surgical treatment in children treated for diaphyseal forearm fractures.

We found a significantly higher refracture risk for CR and nonoperatively treated patients than previously reported [5,6,11,14-16]. Refracture rate after nonoperative treatment has been reported as being as low as 1% [20]. The increased risk of refracture and malunion in the nonoperative group compared with FIN treated patients has not been reported before to our knowledge.

It could be speculated that casting time was too short as the majority of refractures occurred within the first 3 months after the initial fracture. Mean casting time in the nonoperative group was 36 days and in the CR group 32 days, which is relatively short compared with cast immobilization of 43 days followed by 34 days of bracing in the study of Tisosky et al. [20].

In concordance with current literature, malunion was significantly lower for FIN patients than for nonoperative patients. Our definition of malunion can be debated and may not reflect the functional outcome. What matters to patients is the risk of refracture, functional outcome, and cosmetic aspects. Unfortunately, our data does not provide information concerning patient-reported outcomes or the range of motion—supination and pronation, in particular. We are thus not able to verify the clinical significance of our malunion definition. However, we found a high occurrence of malunion in the nonoperative and CR groups. This indicates that FIN treatment should be considered, as angulations above 10° may limit rotation [21]. It could be speculated based on our data that malunion is associated with increased refracture risk or maybe the nail itself protects against early refracture. Prospective studies are warranted to investigate these matters.

Overall, 16% of non-surgical patients were converted to FIN, which is lower than previously reported with up to 30% of conversion rate in the study by Sinikumpu et al. [6]. The lower conversion rate in our study may be explained by the high number of patients receiving initial FIN treatment. In this way, patients with high risk of secondary dislocation may have been initially treated with FIN, thus reducing the need for secondary FIN surgery.

In the FIN group, 1% of patients had secondary surgery due to infection. We do not have any reliable data on superficial infections, or tendon or nerve complications as these may not have required a second surgical procedure and may be underreported in patient charts. Likewise, complications of casting may be underreported as well. Therefore, our study cannot provide any firm recommendations on treatment choice but merely serves as informative for comparative prospective studies, where complications are systematically registered and graded by severity.

### Generalizability

The validation of diagnoses and the identification of 10% of cases as misclassified may in part explain the significantly higher proportion of FIN-treated patients in our study population (87%) compared with a newly published Danish register study that documented an invasive treatment proportion of 23% [7]. That study acknowledged that validity of the diagnostic codes may have been low and especially that many distal forearm fractures may have been falsely included. Thus, we believe that our high treatment proportion of diaphyseal forearm fractures treated with FIN nails more accurately reflects the clinical reality at our participating institutions. However, this is a very high rate compared with the 8–70% reported in the literature in diaphyseal fractures [5,6,8,13]. The preference for surgical treatment among the treating institutions in our study may be explained by the fact that pediatric forearm fractures are often not treated by pediatric orthopedic surgeons, but by on-call non-specialists with insufficient knowledge and skills in pediatric nonoperative fracture treatment. The participating institutions thus seem to follow the international tendency of an increased operative treatment rate for pediatric diaphyseal forearm fracture [7,22].

### Limitations

We did not investigate other complications related to surgical treatment such as nerve damage or tendon rupture and why the full picture of FIN is warranted. The treatment decision was at the discretion of the surgeons having all 3 treatment options available and not on a rigid treatment algorithm. This implies that less severe fractures were more likely to be treated either nonoperatively or by means of CR instead of FIN. Theoretically this should mitigate the refracture risk instead of increasing it as seen in our study.

We did not investigate the quality of the casts and therefore high malunion rates may be explained by poor casting technique.

We do not expect patient migration in the study period and it is unlikely to have influenced the refracture rate.

Another bias could have been the significant difference in age between the treatment groups. Other factors could have introduced bias in our material, such as comorbidities, surgeon experience, or fracture severity.

In the comparison of malunion between treatment groups, we defined malunion based on measurements performed at the last available radiograph during follow-up. While this reflects the available real-life clinical data, the interval between primary and final radiograph differed significantly between the surgically and non-surgically treated groups. This information bias might lead to an overestimation of the malunion rates for non-surgically treated patients

## Conclusion

The risk of refracture and malunion of diaphyseal forearm fractures in children was significantly higher for nonoperative treatment and closed reduction compared with FIN treatment. Infection requiring secondary surgery was reported in the FIN group as a rare complication.

*In perspective,* our findings are concerning as they may indicate that the skills required for effective nonoperative management, such as proper casting techniques, may be diminishing. This shift could inadvertently make surgical intervention appear to be the more convenient and reliable option, particularly as casting techniques are no longer a core component of the orthopedic training curriculum for on-call surgeons.

## Notes

Conceptualization: all authors. Methodology: OR, PHG, SK, JDR, and AA. Formal analysis: HCH. Investigation: OR, PHG, HCB, SK, JDR, and AA. Data curation: all authors. Writing—original draft: HCH and OR. Writing—review and editing: all authors. Supervision: AA. Project administration: OR, PHG, SK, JDR, and AA.

Handling co-editors: Cecilia Rogmark and Robin Christensen

*Acta* thanks Vera Baasland Halvorsen and Ebba Fridh for help with peer review of this manuscript.

## References

[1] Caruso G, Caldari E, Sturla F D, Caldaria A, Re D L, Pagetti P, et al. Management of pediatric forearm fractures: what is the best therapeutic choice? A narrative review of the literature. Musculoskelet Surg 2021; 105(3): 225-34. doi: 10.1007/s12306-020-00684-6. 33058085 PMC8578082

[2] Fuller D J, McCullough C J. Malunited fractures of the forearm in children. J Bone Joint Surg Br 1982; 64(3): 364-7. doi: 10.1302/0301-620X.64B3.7096406. 7096406

[3] Bowman E N, Mehlman C T, Lindsell C J, Tamai J. Nonoperative treatment of both-bone forearm shaft fractures in children. J Pediatr Orthop 2011; 31(1): 23-32. doi: 10.1097/BPO.0b013e318203205b. 21150728 PMC3073825

[4] Noonan K J, Price C T. Forearm and distal radius fractures in children. J Am Acad Orthop Surg 1998; 6(3): 146-56. doi: 10.5435/00124635-199805000-00002. 9689186

[5] Leuba A, Ceroni D, Tabard-Fougère A, Lutz N. Clinical and financial impacts of flexible intramedullary nailing in pediatric diaphyseal forearm fractures: a case-control study. J Child Orthop 2022; 16(3): 220-6. doi: 10.1177/18632521221106380 35800656 PMC9254019

[6] Sinikumpu J J, Lautamo A, Pokka T, Serlo W. Complications and radiographic outcome of children’s both-bone diaphyseal forearm fractures after invasive and non-invasive treatment. Injury 2013; 44(4): 431-6. doi: 10.1016/j.injury.2012.08.032. 22986071

[7] Hansen R T, Borghegn N W, Gundtoft P H, Nielsen K A, Balslev-Clausen A, Viberg B. Change in treatment preferences in pediatric diaphyseal forearm fractures: a Danish nationwide register study of 36,244 fractures between 1997 and 2016. Acta Orthop 2023; 94: 32-7. doi: 10.2340/17453674.2023.7132. 36727711 PMC9893835

[8] Flynn J M, Jones K J, Garner M R, Goebel J. Eleven years experience in the operative management of pediatric forearm fractures. J Pediatr Orthop 2010; 30(4): 313-19. doi: 10.1097/BPO.0b013e3181d98f2c. 20502228

[9] Lyman A, Wenger D, Landin L. Pediatric diaphyseal forearm fractures: epidemiology and treatment in an urban population during a 10-year period, with special attention to titanium elastic nailing and its complications. J Pediatr Orthop B 2016; 25(5): 439-46. doi: 10.1097/BPB.0000000000000278. 26919620

[10] Zilliacus K, Nietosvaara Y, Helenius I, Laaksonen T, Ahonen M, Grahn P. The risk of nerve injury in pediatric forearm fractures. J Bone Jt Surg 2023; 105(14): 1080-6. doi: 10.2106/JBJS.22.01392. 37141456

[11] Peterlein C D, Modzel T, Hagen L, Ruchholtz S, Krüger A. Long-term results of elastic-stable intramedullary nailing (ESIN) of diaphyseal forearm fractures in children. Medicine (Baltimore) 2019; 98(11): e14743. doi: 10.1097/MD.0000000000014743. 30882642 PMC6426625

[12] Lieber J, Dietzel M, Scherer S, Schäfer J F, Kirschner H J, Fuchs J. Implant removal associated complications after ESIN osteosynthesis in pediatric fractures. Eur J Trauma Emerg Surg 2022; 48(5): 3471-8. doi: 10.1007/s00068-021-01763-4. 34338820 PMC9532316

[13] Cruz A I, Kleiner J E, DeFroda S F, Gil J A, Daniels A H, Eberson C P. Increasing rates of surgical treatment for paediatric diaphyseal forearm fractures: a National Database Study from 2000 to 2012. J Child Orthop 2017; 11(3): 201-9. doi: 10.1302/1863-2548.11.170017. 28828064 PMC5548036

[14] Korhonen L, Perhomaa M, Kyrö A, Pokka T, Serlo W, Merikanto J, et al. Intramedullary nailing of forearm shaft fractures by biodegradable compared with titanium nails: results of a prospective randomized trial in children with at least two years of follow-up. Biomaterials 2018; 185: 383-92. doi: 10.1016/j.biomaterials.2018.09.011. 30292588

[15] Syed A N, Ashebo L, Lawrence J T R. Refracture following operative treatment of pediatric both bone forearm fractures. J Pediatr Orthop. Published online October 31, 2023. doi: 10.1097/BPO.0000000000002552. 37904588

[16] Han B, Wang Z, Li Y, Xu Y, Cai H. Risk factors for refracture of the forearm in children treated with elastic stable intramedullary nailing. Int Orthop 2019; 43(9): 2093-7. doi: 10.1007/s00264-018-4184-4. 30280215

[17] von Elm E, Altman D G, Egger M, Pocock S J, Gøtzsche P C, Vandenbroucke J P. The Strengthening the Reporting of Observational Studies in Epidemiology (STROBE) statement: guidelines for reporting observational studies. J Clin Epidemiol 2008; 61(4): 344-9. doi: 10.1016/j.jclinepi.2007.11.008. 18313558

[18] Price C T, Scott D S, Kurzner M E, Flynn J C. Malunited forearm fractures in children. J Pediatr Orthop 1990; 10(6): 705-12. doi: 10.1097/01241398-199011000-00001. 2250053

[19] Christensen R, Ranstam J, Overgaard S, Wagner P. Guidelines for a structured manuscript: statistical methods and reporting in biomedical research journals. Acta Orthop 2023; 94: 243-9. doi: 10.2340/17453674.2023.11656. 37170796 PMC10176201

[20] Tisosky A J, Werger M M, McPartland T G, Bowe J A. The factors influencing the refracture of pediatric forearms. J Pediatr Orthop 2015; 35(7): 677-81. doi: 10.1097/BPO.0000000000000355. 25436481

[21] Tarr R R, Garfinkel A I, Sarmiento A. The effects of angular and rotational deformities of both bones of the forearm: an in vitro study. J Bone Joint Surg Am 1984; 66(1): 65-70. http://www.ncbi.nlm.nih.gov/pubmed/6690445. 6690445

[22] Ömeroğlu H, Cassiano Neves M. Tendency towards operative treatment is increasing in children’s fractures: results obtained from patient databases, causes, impact of evidence-based medicine. EFORT Open Rev 2020; 5(6): 347-53. doi: 10.1302/2058-5241.5.200012. 32655890 PMC7336186

